# Genetic Analysis of the *Gdh* and *Bg* Genes of Animal-Derived *Giardia duodenalis* Isolates in Northeastern China and Evaluation of Zoonotic Transmission Potential

**DOI:** 10.1371/journal.pone.0095291

**Published:** 2014-04-18

**Authors:** Aiqin Liu, Fengkun Yang, Yujuan Shen, Weizhe Zhang, Rongjun Wang, Wei Zhao, Longxian Zhang, Hong Ling, Jianping Cao

**Affiliations:** 1 Department of Parasitology, Harbin Medical University, Harbin, Heilongjiang, China; 2 National Institute of Parasitic Diseases, Chinese Center for Disease Control and Prevention, Key Laboratory of Parasite and Vector Biology, Ministry of Health, WHO Collaborating Centre for Malaria, Schistosomiasis and Filariasis, Shanghai, China; 3 College of Animal Science and Veterinary Medicine, Henan Agricultural University Zhengzhou, Henan, China; Obihiro University of Agriculture and Veterinary Medicine, Japan

## Abstract

**Background:**

*Giardia duodenalis* is a common intestinal parasite that infects humans and many other mammals, mainly distributing in some areas with poor sanitation. The proportion of the human giardiasis burden attributable to *G. duodenalis* of animal origin differs in different geographical areas. In Mainland China, genetic data of the *gdh* and *bg* genes of *G. duodenalis* from animals are only limited in dogs and cats. The aim of the study was to provide information on the genetic characterizations of animal-derived *G. duodenalis* isolates (from rabbits, sheep and cattle) at both loci in Heilongjiang Province, Northeastern China, and to assess the potential for zoonotic transmission.

**Methodology/Principal Findings:**

61 *G. duodenalis* isolates from animal feces (dairy and beef cattle, sheep and rabbits) in Heilongjiang Province were characterized at the *gdh* and *bg* loci in the present study. The *gdh* and *bg* gene sequences of sheep-derived *G. duodenalis* assemblage AI, and the *gdh* sequences of rabbit-derived *G. duodenalis* assemblage B had 100% similarity with those from humans, respectively. Novel subtypes of *G. duodenalis* were identified, with one and seven subtypes for assemblages A and E at the *gdh* locus, and two and three subtypes for assemblages B and E at the *bg* locus, respectively. Three pairs of the same *bg* sequences of assemblage E were observed in sheep and cattle.

**Conclusions/Significance:**

This is the first description of genetic characterizations of the *gdh* and *bg* genes of *G*. *duodenalis* from rabbits, sheep and cattle in Mainland China. Homology analysis of assemblages AI and B implied the possibility of zoonotic transmission. The novel subtypes of assemblages of *G*. *duodenalis* may represent the endemic genetic characteristics of *G. duodenalis* in Heilongjiang Province, China.

## Introduction

*Giardia duodenalis* (syn. *G. lamblia*, *G. intestinalis*) is an intestinal, parasitic, flagellate protozoan that infects humans as well as a wide variety of domestic animals and wildlife species. Giardiasis is one of the major neglected tropical diseases occurring in rural areas and poor urban areas. Human giardiasis has complicated clinical manifestations, ranging from the absence of symptoms to acute or chronic diarrhea, abdominal pain, nausea, vomiting and so on, depending on the health status of hosts, and the dose and virulence of the parasites [1]. It has been shown that *G. duodenalis* is a complex species, with eight genetically distinct assemblages (A-H) having been identified; each of them has significant differences in host range or host specificity [2], [3]. Assemblages A and B have the broadest host specificity, and are found to infect humans and many other mammals. However, the other assemblages are mostly found in non-human mammals, with assemblages C and D in dogs, assemblage E in hoofed livestock, assemblage F in cats, assemblage G in rodents, and assemblage H in marine mammals [3], [4]. In fact, the majority of cases of human giardiasis are caused by assemblages A and B, with assemblages C, D, E and F occasionally isolated from humans [4].

Based on the fact that assemblages A and B in humans have also been found in animals, the role host animals play in the epidemiology of human giardiasis has received increasing attention. The animal-derived sequences of assemblages A and B have been reported to show 100% similarity with those from humans at the triose phosphate isomerase (*tpi*), glutamate dehydrogenase (*gdh*) and β*-*giardin (*bg*) loci; thus, both assemblages are considered to have the possibility of zoonotic transmission [5]–[7]. PCR-based molecular analysis techniques have been used widely to trace the sources of infection or contamination and to assess the human giardiasis burden attributable to animal origin. Currently, several genetic loci are used to identify *Giardia* isolates at the genotype and subtype levels, most commonly including the small subunit ribosomal RNA (SSU rRNA), *bg*, *gdh* and *tpi* genes [8]. *Tpi, gdh* and *bg* genes are common genotyping and subtyping markers of *G. duodenalis*, whereas the SSU rRNA gene is commonly used markers for the species and assemblage differentiation of *Giardia* [9]. Although a single locus has been used in most of epidemiological studies of giardiasis, multilocus genotyping (MLG) tools are used increasingly in characterizing *G. duodenalis* isolates from humans and animals. The data based on MLG tools can greatly improve our understanding of the relationship between humans and animals as hosts and reservoirs, including elucidation of the transmission routes and dynamics of human giardiasis, and assessment of the burden of human giardiasis of animal origin.

In Mainland China, the majority of genetic data of *G. duodenalis* from humans and animals are based on the *tpi* gene and only one report described the genetic characterizations of the *gdh* and *bg* gene of animal-derived *G. duodenalis* conducted in Guangzhou [10]. In the present study, we obtained *gdh* and *bg* gene sequences of *G. duodenalis* isolates from rabbits, sheep and cattle in Heilongjiang Province. The aim of the study was to understand the endemic genetic characterizations of animal-derived isolates of *G. duodenalis* at both loci and to assess its potential for zoonotic transmission in the investigated areas by aligning the obtained sequences with those from GenBank.

## Materials and Methods

### Ethics Statement

The study was conducted in accordance with the guidelines of the Regulations for the Administration of Affairs Concerning Experimental Animals and was approved by the Animal Ethical Committee of Harbin Medical University (HMUIRB20130009). No other specific permits were required for the described field studies. The locations where we sampled are not protected in any way. The field studies did not involve endangered or protected animal species. Before beginning work on the study, we contacted the farm owners and obtained their permission. In this study, all the samples were taken immediately from animal fresh feces deposited on the ground after animal defecation instead of operating or experimenting on the animals. During the procedure, the animals were not hurt at all.

### Source of isolates

*G. duodenalis* isolates were all obtained from animal feces (26 from cattle in seven farms, 21 from sheep in four farms and 14 from rabbits in five farms) in Heilongjiang Province, China between October 2008 and November 2011. Genomic DNA was extracted using a QIAamp DNA Mini Kit (Qiagen, Hilden, Germany) according to the manufacturer's recommendations and DNA preparations were stored at −20°C before PCR analysis. 61 *G. duodenalis* isolates, which were identified based on the *tpi* gene in the previous studies [5], , were randomly chosen as the study subjects of genetic characterizations of the *gdh* and *bg* genes of *G. duodenalis* (Table 1).

**Table 1:** PCR amplification rates of different *G. duodenalis* assemblages at the *gdh* and *bg* loci.

Host	*tpi*	*gdh*		*bg*	
	Assemblages (no.)	Assemblages (no.)	Amplification rate (%)	Assemblages (no)	Amplification rate (%)
rabbit	B (14)	B (10)	71.4	B (12)	85.7
sheep	A (4)	A (4)	100	A (4)	100
	E (17)	E (16)	88.4	E (12)	67.4
cattle	B (10)	E (8)		E (4)	
	E (16)	E (14)		E (13)	
Total	A, B, E (61)	A, B, E (52)	85.2	A, B, E (45)	73.8

### PCR amplification of the *gdh* and *bg* genes of *G. duodenalis*

Each DNA preparation of *G. duodenalis* was characterized using two distinct protocols for nested PCR depending on the target to be amplified. A fragment of approximately 530 bp of the *gdh* gene was amplified using the external primers Gdh1 and Gdh2, and internal primers Gdh3 and Gdh4 [13]. The amplification of 511 bp of the *bg* gene was performed using the external primers G7 and G759 [14], and the internal primers GBF and GBR [6]. PCR products were visualized by electrophoresis in 1.5% agarose gel stained with ethidium bromide. DNA preparations of PCR-negative samples at either or both of the two loci were subjected to three repeated PCR amplifications.

### DNA sequencing and molecular analysis

All secondary PCR products were sequenced with secondary PCR primers at each locus on an ABI PRISMTM 3730 DNA Analyzer (Applied Biosystems, Carlsbad, CA, USA), using a BigDye Terminator v3.1 Cycle Sequencing kit (Applied Biosystems, USA). Accuracy of the sequencing data was confirmed by sequencing in both directions. Nucleotide sequences obtained in the present study were subjected to BLAST searches (http://www.ncbi.nlm.nih.gov/blast/) and were then aligned with *G. duodenalis* reference sequences downloaded from GenBank and analyzed using Clustal X 1.83.

## Results

### PCR amplification of *G. duodenalis* at the *gdh* and *bg* loci

61 *Giardia* DNA preparations were subjected to nested PCR amplification of the *gdh* and *bg* genes. 52 were successfully amplified at the *gdh* locus, with four assemblage A, 10 assemblage B, and 38 assemblage E being identified. A fragment of the *bg* gene of the expected size was obtained out of 45 samples, with four assemblage A, 12 assemblage B, and 29 assemblage E being identified. The PCR amplification rates were 85.2% (52/61) at the *gdh* locus versus 73.8% (45/61) at the *bg* locus, with 100% (4/4) versus 100% (4/4) for assemblage A, 71.4% (10/14) versus 85.7% (12/14) for assemblage B, and 88.4% (38/43) versus 67.4% (29/43) for assemblage E (Table 1).

### Characterization of the *gdh* and *bg* gene sequences of assemblage A

Sequence analysis of the *gdh* gene from four sheep-derived *G. duodenalis* isolates revealed the presence of two subtypes of assemblage A. One subtype (KC960643) (n = 3) was identified as AI, having 100% homology with the human-derived subtype AI sequences (GQ329674 and GQ329676) [15]; the same sequences have been found in seals and cats [3], [16]. The other subtype (KC960644) was not identical to any reported assemblage A sequences.

However, at the *bg* locus, four identical sequences (KC960630) were shown to have 100% similarity with those of human-derived (GQ329671, GQ329672) and animal-derived assemblage AI isolates (alpacas, sheep, ferrets and cattle) [15], [17]–[20].

### Characterization of the *gdh* and *bg* gene sequences of assemblage B

10 out of 14 rabbit-derived *G. duodenalis* isolates were successfully amplified and sequenced at the *gdh* locus, with all of them being identified as assemblage B. Nine were identical to each other (KC960645) and had 100% similarity with the human-derived assemblage B sequence from Brazil (EF507682) [16]. The remaining one (KC960646) had 100% homology with the sequences isolated from humans in Australia (EF685680) and Brazil (EF507646, EF507664, EF507668, EF507671, EF507672), and from chinchillas in Brazil (HM134212-HM134214) [16], [21], [22].

12 *bg* gene sequences of rabbit-derived *G. duodenalis* isolates were successfully obtained and identified as assemblage B. Two novel subtypes were detected with a single base variation between each other, with one representing 10 sequences (KC960631) and the other representing two sequences (KC960632), and both of them have one or two base differences with the published sequence AB618785, respectively.

### Characterization of the *gdh* and *bg* gene sequences of assemblage E

16 out of 17 sheep-derived *G. duodenalis* isolates were successfully amplified and sequenced at the *gdh* locus, belonging to four novel subtypes of assemblage E (KC960647 to KC960650). Four subtypes of assemblage E were observed from 22 *gdh* gene sequences obtained successfully from 26 cattle-derived *G. duodenalis* isolates, with one subtype (KC960651) having the same sequence as those of the cattle-derived isolates (EF507644 and EF507645) [16] and the other three subtypes (KC960652 to KC960654) being novel (Tables 1, 2). Using the sequence EF507645 as a reference sequence, genetic polymorphism was observed with one- to five-base variations at 12 nucleotide sites in the *gdh* nucleotide sequences (Table 2).

**Table 2:** Variations at the *gdh* locus among intra-assemblage E of *G. duodenalis* isolates from sheep and cattle in Heilongjiang Province, China.

Host	Accession no. in GenBank	Nucleotide at position											
		95	97	105	116	141	172	210	272	310	313	324	455
	EF507645(ref)	T	T	T	C	A	C	G	T	G	C	T	G
sheep	KC960647	T	T	C	C	A	C	G	T	G	C	T	G
	KC960648	T	T	C	C	A	C	G	T	G	C	T	A
	KC960649	T	T	C	C	G	C	G	T	A	C	T	G
	KC960650	T	T	C	C	A	C	A	T	A	T	T	A
cattle	KC960651	T	T	T	C	A	C	G	T	G	C	T	G
	KC960652	C	T	T	C	A	C	G	T	G	C	T	G
	KC960653	C	T	T	A	A	C	G	G	G	C	T	G
	KC960654	C	C	T	C	A	T	G	T	G	C	C	G

At the *bg* locus, five subtypes of assemblage E were found among 12 successful sequences obtained from sheep-derived *G. duodenalis* isolates. Three subtypes (KC960633 to KC960635) have been described previously in sheep, goats and cattle [18], [23]–[26] and the remaining two subtypes (KC960636 and KC960637) were novel. 17 *bg* sequences of cattle-derived *G. duodenalis* isolates belonged to five subtypes, with four subtypes (KC960638 to KC960641) having been reported in sheep, goats and cattle [8], [18], [23]–[26] and the remaining one (KC960642) being novel (Tables 1, 3). Using the sequence EU726980 as a reference sequence, genetic polymorphism was observed with one- to three-base variations at seven nucleotide sites in the *bg* nucleotide sequences (Table 3).

**Table 3:** Variations at the *bg* locus among intra-assemblage E of *G. duodenalis* isolates from sheep and cattle in Heilongjiang Province, China.

Host	Accession no. in GenBank	Nucleotide at position						
		40	41	58	100	163	264	408
	EU726980(ref)	A	G	C	C	A	C	T
sheep	KC960633	A	G	C	C	A	C	T
	KC960634	A	G	T	C	A	C	C
	KC960635	A	G	T	C	G	C	C
	KC960636	G	A	C	C	A	C	C
	KC960637	A	G	T	T	G	C	T
cattle	KC960638	A	G	T	C	A	C	C
	KC960639	A	G	C	C	A	C	T
	KC960640	A	G	C	C	A	C	C
	KC960641	A	G	T	C	G	C	C
	KC960642	A	G	T	C	A	T	C

It was also observed that three sheep-derived *bg* gene sequences of assemblage E (KC960634, KC960633 and KC960635) showed 100% homology with the three from cattle (KC960638, KC960639 and KC960641), respectively (Table 3).

## Discussion

PCR-based techniques have been used widely for the identification, population genetics and epidemiology of *G. duodenalis* in humans, animals and environmental samples. MLG analysis is increasingly used for the characterizations of *G. duodenalis* isolates and assessment of human disease burden caused by zoonotic transmission. *G. duodenalis* isolates from different animal origins were characterized at the *gdh* and *bg* loci in the present study. The *gdh* gene sequence (KC960643) representing three sheep-derived assemblage AI isolates has been described in human giardiasis cases in Sweden [15]. Meanwhile, all the four assemblage AI isolates from sheep were identical to each other (KC960630) and had 100% similarity at the *bg* locus with a human-derived *G. duodenalis* isolate from Sweden [15]. To date, identification of genetic variations of nucleotide sequences within assemblage A at the *gdh*, *bg* and *tpi* loci has led to the designation of assemblages AI, AII and AIII, which all appear to have host specificity. Assemblage AI is commonly found in animals and is sometimes seen in humans; assemblage AII mainly infects humans and is occasionally detected in animals; assemblage AIII has circulated in wildlife [27]. Recently, assemblage AIV was designated based on the base differences of the *tpi* gene sequence compared to AI, AII and AIII [5]. In the present study, the assemblage AI sequences at the *gdh* and *bg* loci showed 100% homology with those from humans in Sweden [15]. The result above implied that sheep infected with assemblage AI pose a significant threat to local inhabitants and are of public health importance. Unfortunately, no genetic data of human-derived *G. duodenalis* isolates have been obtained in the investigated areas although human giardiasis cases were reported early in 1990 [28]. Even in China, there are also only a few reports about molecular identification of human-derived *G. duodenalis* isolates [29]–[32]. Thus, the transmission routes and dynamics of subtype AI and the human giardiasis burden attributable to *G. duodenalis* of animal origin remain to be clarified by epidemiological data of human and animal giardiasis cases.

Assemblage B is one of the two major assemblages causing human giardiasis with a broad animal host range. It has been isolated from cattle, sheep, pigs, horses, dogs, cats, and rabbits [27]. Two subtypes (KC960645 and KC960646) were identified in the present study by analyzing 10 *gdh* gene sequences from rabbits and both of them had been reported in human giardiasis cases from Brazil and Australia [16], [21]. The result indicated a high potential for cross-species transmission of assemblage B between rabbits and humans due to the similar genetic backgrounds. So far, the strongest evidence supporting transmission from rabbits to humans was based on the report of a foodborne outbreak of giardiasis associated to a family party, where the food preparer, his child, guests, and a pet rabbit were positive for *Giardia* cysts [33]. However, these *Giardia* isolates were not genotyped or subtyped. The outbreak emphasized not only the possibility of domestic-animal-to-person transmission of *G. duodenalis* but also the importance of good hygienic practices in food preparation to prevent the spread of animal-derived *G. duodenalis* cysts in humans. In fact, an early case-control study in eastern England showed an association of giardiasis with exposure to farm animals and pets, particularly pigs, dogs, and cats [34]. In southwestern England, a visit to a farm led to a high frequency of giardiasis among case patients, but none of the associations with specified animal exposures (dogs, cats, horses, cows, sheep) was statistically significant [35].

Assemblage E is mainly found in cloven-hoofed domestic mammals (cattle, water buffaloes, sheep, goats, and pigs [27]. In addition, it has been occasionally reported in cats [4], [8], [36]. The prevalence of assemblage E in cattle was reported to be high up to 75.1% (422/562) in a European study of *G*. *duodenalis* [4]. High occurrence of *G*. *duodenalis* has also been described in goats and sheep in Spain, with a prevalence of 100% (39/39) for goats and 98.7% (74/75) for sheep [18], [23]. By DNA sequence analysis within the partial *tpi*, *gdh* and *bg* genes, the genetic polymorphism of assemblage E has been observed [5], [8], [12], [18], [25], [37], [38]. In the present study, the alignment result of the *gdh* gene sequences showed the presence of seven novel subtypes of assemblage E, four from sheep (KC960647 to KC960650) and three from cattle (KC960652 to KC960654) (Table 2). At the *bg* locus, five subtypes were observed among sheep-derived and cattle-derived *G. duodenalis* assemblage E isolates, respectively, with three novel subtypes (KC960636, KC960637, KC960642) being found in total. The identification of three pairs of the same *bg* sequences from sheep and cattle (KC960634 versus KC960638, KC960633 versus KC960639 and KC960635 versus KC960641) indicated the possibility of cross-species transmission of *G. duodenalis* E between cloven-hoofed animals in the investigated areas (Table 3).

In the present study, we observed the phenomenon of “assemblage-swapping” (different assemblages at different loci in the same isolate. Cattle-derived *G. duodenalis* isolates were identified as assemblage E based on successful PCR and sequencing analysis, respectively at the *gdh* locus (n = 8) and at the *bg* locus (n = 4); however, all the DNA preparations used above were previously identified as assemblage B based on the *tpi* gene (Table 1). Similar findings were reported in an identification study of dog-derived *G. duodenalis* isolates, where an isolate was typed as assemblage AI based on the *tpi* gene while it was typed as assemblage D based on the *gdh* and *b*g genes [39]. It has also been observed that three out of five human-derived *G. duodenalis* isolates were identified as assemblage A at the18S rRNA locus and assemblage B at the *gdh* locus, while the remaining two were identified as assemblage B at the 18S rRNA locus and assemblage A at the *gdh* locus [40]. A recent multi-locus genotyping identified 41 human-derived *G. duodenalis* isolates as assemblage B and 13 as assemblage A based on *tpi, gdh* and *bg* genes, whereas 14 showed multiple assemblage depending on the marker loci [41]. “Assemblage-swapping” in the diagnosis of *G. duodenalis* infection may be related to mixed assemblage infections in the fecal samples or recombination between assemblages.

MLG is a powerful tool for tracing the infection or contamination source of *G. duodenalis* and assessing the zoonotic potential of giardiasis. In the present study, homology analysis showed that two *gdh* sequences of rabbit-derived assemblage B isolates (KC960645 and KC960646) were identical to those from humans. However, at the *bg* locus, no sequences had 100% similarity with those from the cases of human giardiasis. The results indicated that single gene nucleotide analysis of *G. duodenalis* isolates from animals and humans at the genotype and subtype levels are insufficient for assessment of the disease burden of zoonotic transmission.

In the present study, PCR was observed to have higher amplification rate at the *gdh* locus (85.2%, 52/61) than at the *bg* locus (73.8%, 45/61), which might be mainly related to the primers and genetic structure of target fragments, for the other factors influencing PCR efficiency were identical such as the same quality and quantity of DNA templates and the quality and characterization of DNA polymerase used. Even though the primers were designed to bind ‘conserved’ regions in the amplified genes, excessive mismatches in the binding regions of primer sequences might result in the failure of PCR amplification for some isolates of *G. duodenalis.* In fact, numerous molecular data have confirmed both inter- and intra-assemblage genetic variations [27]. Variable base substitution rates of *G. duodenalis* have been reported at different genetic loci [9]. Such differences in nucleotide sequences, especially base variations in primer binding sites, might lead directly to the failure in the primer-template binding in some *G. duodenalis* isolates. PCR analysis by Scorza et al showed positive amplification of the *gdh*, *bg*, and *tpi* genes in 91.0% (172/189), 84.8% (145/171) and 19.8% (34/172) of *G. duodenalis* cyst-positive samples from the mammalian feces, respectively [39]. PCR amplification rates were 100% (63/63), 61.9% (39/63) and 56.0% (14/25), respectively at the *gdh*, *bg* and *tpi* loci in a study of molecular characterization of *Giardia* isolates from clinical infections following a waterborne outbreak [42]. In another comparative study of the *tpi* and *gdh* genes for detection and genotyping of human-derived *G. duodenalis* isolates, the *tpi* gene was amplified from 96.2% (25/26) of samples, whereas only 81% (21/26) were positive when the *gdh* gene was targeted [43]. It was also observed that PCR amplification rates differed between different assemblages. Assemblage A had the highest amplification rate (100%) at either the *gdh* locus or the *bg* locus, followed by assemblage E (88.4%) and assemblage B (71.4%) at the *gdh* locus versus assemblage B (85.7%) and assemblage E (67.4%) at the *bg* locus (Table 1). In general, no matter which gene was amplified, assemblage A had higher amplification rate than assemblage B. This result might be related to the fact that assemblage B exhibits higher allelic sequence heterogeneity and genetic recombination compared to assemblage A [27], [4], [44], [45]. In a recent study, both intra- and inter-assemblage recombination and meiotic sex were seen from assemblages A to G by analyzing two or more genes of *G. duodenalis* isolates from humans and other animals [21]. Thus, the reasons for different PCR amplification rates of different assemblages at different loci need to be elucidated by more systematic and more complete genetic data from a large number of *G. duodenalis* isolates in the future.

The present study is the first report about genetic characterizations of the *gdh* and *bg* genes of *G*. *duodenalis* isolates from rabbits, sheep and cattle in Mainland China. In conclusion, the obtained data provide useful information for further genotyping or subtyping studies of *G. duodenalis*. The findings of animal-derived assemblages AI and B having 100% homology with human-derived *G. duodenalis* isolates at the *gdh* and *bg* loci imply the possibility of zoonotic transmission in the investigated areas. The identification of the novel subtypes of assemblages AI, B and E based on the *gdh* and *bg* genes might reflect the characteristic geographical distribution of *G. duodenalis*. To better understand the transmission dynamics of *G. duodenalis* and assess the burden of human giardiasis caused by animals, molecular epidemiological studies of giardiasis should be conducted in humans and animals living in the same household or localized focus of endemicity.
